# Sustained virological response in HIV/HCV co-infected patients without rapid virological response (RVR) on peginterferon-ribavirin therapy

**DOI:** 10.1186/1758-2652-13-S4-P208

**Published:** 2010-11-08

**Authors:** P Labarga, ME Vispo, JM Guardiola, C Miralles, L Martín-Carbonero, J Portu, P Barreiro, P Miralles, J Morello, MJ Tellez, P Bancalero, V Asensi, NI Rallón, I Santos, L Morano, K Aguirrebengoa, MJ Rios, R Rubio, K Neukam, J González-Lahoz, V Soriano

**Affiliations:** 1 Hospital Carlos III, Sinesio Delgado 10, Madrid, Spain; 2 Hospital San Pablo, Barcelona, Spain; 3 Hospital Xeral-Cies, Pontevedra, Spain; 4 Hospital Xagorrichu, Vitoria, Spain; 5 Hospital Gregorio Marañón, Madrid, Spain; 6 Hospital Clinico San Carlos, Madrid, Spain; 7 Hospital de Jerez, Jerez, Spain; 8 Hospital GeneralAsturias, Oviedo, Spain; 9 Hospital de La Princesa, Madrid, Spain; 10 Hospital Meixoeiro, Vigo, Spain; 11 Hospital de Cruces, Bilbao, Spain; 12 Hospital Virgen de la Macarena, Sevilla, Spain; 13 Hospital 12 de Octubre, Madrid, Spain; 14 Hospital de Valme, Sevilla, Spain

## Background

Undetectable HCV-RNA at week 4 of therapy (RVR) is one of the best predictors of SVR in patients with chronic hepatitis C. However, there is scarce information about the variables influencing the achievement of SVR in patients without RVR.

## Methods

A prospective study in which HIV-HCV coinfected patients were randomized to receive either pegylated IFNα-2a 180 µg/week plus ribavirin (RBV) at two different doses: i) 1000-1200 mg/day (arm A) or ii) 2000 mg/day plus erythropoietin (EPO) from baseline to week 4 (arm B), was conducted. Patients not achieving RVR prolonged therapy to 48 weeks for G2-3 and to 72 for G1-4. Liver fibrosis was measured using transient elastometry, being liver stiffness values ≥9.5 kPa as reflect of Metavir ≥F3. RBV trough concentrations at week 4 and pharmacogenetic studies including polymorphisms at the ITPA, ENT1 and IL28B genes were also performed. Variables associated to SVR in those not achieving RVR were examined.

## Results

A total of 108 patients had reached the end of follow-up at the time the analysis (82% males; mean age, 43 years; mean baseline HCV-RNA, 6.4 log IU/mL; 91% HCV G1-4; 50% METAVIR ≥F3 estimates; mean CD4 count, 566 cells/µL; 93% on HAART and 84% with plasma HIV-RNA <50 copies/mL). In the on-treatment analysis 33% achieved SVR (31% vs 60% for G1-4 and G2-3; p<0.05; 42% vs 23% for patients with Hb drop at week 4 ≥2 vs <2 g/dL; p=0.04; 26% vs 43% for ≥F3 vs <F3; p=0.12; 57% vs 14% for patients with undetectable vs detectable HCV-RNA at week 12; p<0.001; 59% vs 18% for patients completing vs non-completing treatment prolongation; p<0.001). No differences in SVR rates were found comparing arms A vs B. Mean baseline HCV-RNA in patients with vs without SVR were 6.2 vs 6.5 log IU/mL, respectively [p=0.02]. The SVR rate was 57% vs 25% in IL28B CC vs CT/TT carriers [p=0.04]. No significant differences were found for other genetic polymorphisms examined. In the multivariate analysis (OR [95% CI], p) undetectable HCV-RNA at week 12 (5 [2-14], 0.001) and completing treatment prolongation (4 [1.5-12], 0.005) were the only independent predictors of SVR. IL28B CC was not included in the final analysis as it was only available for 42 patients.

## Conclusions

Optimizing duration of HCV therapy (48 weeks for G2-3 and 72 weeks for G1-4) in HIV/HCV co-infected patients without RVR can clearly improve the SVR rate. Moreover, achievement of undetectable HCV-RNA at week 12 is a strong predictor of SVR in this population.

